# Correlation Between Mammographic Radiomics Features and the Level of Tumor-Infiltrating Lymphocytes in Patients With Triple-Negative Breast Cancer

**DOI:** 10.3389/fonc.2020.00412

**Published:** 2020-04-15

**Authors:** Hongwei Yu, Xianqi Meng, Huang Chen, Xiaowei Han, Jingfan Fan, Wenwen Gao, Lei Du, Yue Chen, Yige Wang, Xiuxiu Liu, Lu Zhang, Guolin Ma, Jian Yang

**Affiliations:** ^1^Department of Radiology, China-Japan Friendship Hospital, Beijing, China; ^2^Beijing Engineering Research Center of Mixed Reality and Advanced Display, School of Optics and Photonics, Beijing Institute of Technology, Beijing, China; ^3^Department of Pathology, China-Japan Friendship Hospital, Beijing, China; ^4^Graduate School of Peking Union Medical College, Chinese Academy of Medical Sciences and Peking Union Medical College, Beijing, China; ^5^Department of Science and Education, Shangluo Central Hospital, Shangluo, China

**Keywords:** breast cancer, triple-negative breast cancer, tumor-infiltrating lymphocytes, mammogram, radiomics

## Abstract

**Objectives:** Tumor-infiltrating lymphocytes (TILs) have been identified as a significant prognostic indicator of response to neoadjuvant therapy and immunotherapy for triple-negative breast cancer (TNBC) patients. Herein, we aim to assess the association between TIL levels and mammographic features in TNBC patients.

**Methods:** Forty-three patients with surgically proven TNBC who underwent preoperative mammography from January 2018 to December 2018 were recruited. Pyradiomics software was used to extract 204 quantitative radiomics features, including morphologic, grayscale, and textural features, from the segmented lesion areas. The correlation between radiological characteristics and TIL levels was evaluated by screening the most statistically significant radiological features using Mann–Whitney *U*-test and Pearson correlation coefficient. The patients were divided into two groups based on tumor TIL levels: patients with TIL levels <50% and those with TIL levels ≥50%. The correlation between TIL levels and clinicopathological characteristics was assessed using the chi-square test or Fisher's exact test. Mann–Whitney *U*-test and Pearson correlation coefficient were used to analyze the statistical significance and Pearson correlation coefficient of clinical pathological features, age, and radiological features.

**Results:** Of 43 patients, 32 (74.4%) had low TIL levels and 11 (25.6%) had high TIL levels. The histological grade of the low TIL group was higher than that of the high TIL group (*p* = 0.043). The high TIL group had a more negative threshold Ki-67 level (<14%) than the low TIL group (*p* = 0.017). The six most important radiomics features [uniformity, variance, grayscale symbiosis matrix (GLCM) correlation, GLCM autocorrelation, gray level difference matrix (GLDM) low gray level emphasis, and neighborhood gray-tone difference matrix (NGTDM) contrast], representing qualitative mammographic image characteristics, were statistically different (*p* < 0.05) among the low and high TIL groups. Tumors in the high TIL group had a more non-uniform density and a smoother gradient of the tumor pattern than the low TIL group. The changes in Ki-67, age, epidermal growth factor receptor, radiomic characteristics, and Pearson correlation coefficient were statistically significant (*p* < 0.05).

**Conclusion:** Mammography features not only distinguish high and low TIL levels in TNBC patients but also can act as imaging biomarkers to enhance diagnosis and the response of patients to neoadjuvant therapies and immunotherapies.

## Introduction

Triple-negative breast cancer (TNBC), which is a type of invasive breast cancer, is characterized by severe disease progression, poor prognosis, high recurrence rate, and short survival. Its prognosis varies with clinical, pathologic, and genetic factors (1, 2). Tumor-infiltrating lymphocytes (TILs) reflect an individual's immune tumor response. TIL levels are higher in highly proliferating tumors, including human epidermal growth factor receptor 2 (HER2)-positive and TNBC (3). TILs have a strong prognostic and predictive significance, and high TIL levels are positively correlated with pathological complete response rate and patient survival rate (4–7).

Mammography is the first screening method for breast cancer, especially for women over the age of 45. Findings of typical breast cancer screening mammography include architectural distortion, mass, calcification, asymmetrical breast tissue, and adenopathy (8). Radiomics is different from traditional methods in that it does not use medical images for visual interpretation but instead converts digital medical images into minable data through high-throughput extraction based on various quantitative features such as shape, intensity, size, or volume (9, 10). Radiomics can provide additional information for the diagnosis, prognosis, and prediction in clinical practice (11, 12). Certain qualitative imaging features obtained *via* mammography, breast magnetic resonance imaging (MRI), and ultrasound have been indicated to be correlated with the diagnosis, prognosis, molecular subtyping, and prediction of the response to treatment in breast cancer patients (13–17). Recently, a correlation between dynamic contrast-enhanced magnetic resonance imaging (DCE-MRI) and TIL levels was reported in MRI computer-aided detection of TNBC patients (18). However, breast MRI is expensive and is not widely applied, especially in less developed countries. In contrast, mammography is widely used for screening and diagnosis of breast cancer because of its cost-effectiveness.

Quantitative features of radiomics can distinguish between TNBC and non-TNBC in mammograms, as has been shown in some studies (19, 20). Recently, the relationship between mammographic radiomic features and molecular subtypes of breast cancer was evaluated, which showed that quantitative radiomics imaging features were associated with breast cancer subtypes (21). However, no studies have explored the relationship between TIL levels and the characteristics of mammograms of TNBC patients. Preoperative assessment of TILs is a significant indicator for prognosis and therapy response. In this study, we aimed to investigate the relationship between radiomics imaging characteristics of TNBC patients and TIL levels using radiological methods.

## Materials and Methods

### Patients and Imaging Dataset

The Institutional Ethics Review Committee of the China-Japan Friendship Hospital approved this retrospective study, and informed consent was obtained from all patients. A total of 43 TNBC patients aged 24–87 years (mean age 52.3 years) were included in the analysis. The TNBC patients received preoperative mammograms between January and December 2018. All mammograms were obtained using the Hologic Lorad Selenia digital mammography system (Hologic gen-probe, San Diego, USA). The quantization was set to 14-bit for the full-field digital mammographic images with pixel sizes of 70 μm × 70 μm. Images of the craniocaudal (CC) view and the mediolateral oblique (MLO) view were obtained from mammograms of each patient. A total of 86 mammographic images were analyzed.

### Radiomics Feature Extraction

An experienced breast imaging professional radiologist manually outlined tumor edges in each image of each patient in the TIK-SNAP software (version 3.8, Philly, PA, USA) and extracted the radiological features of the lesion area. The segmentation methods were as follows: (1) import the breast tumor images with DICOM format into the home page by pressing the “Open main image” button; (2) select “Browse” tool and then click “Next” to go to the current image in the “Main Toolbar” drop-down menu; (3) select “Polygon Mode” to manually draw the region of interest (ROI) along the tumor margin; (4) click “Save Segmentation Image” to save the segmentation images into the destination folder in “nii.gz” format. A total of 204 quantitative radiomics features were extracted using Pyradiomics software (version 2.2.0, Boston, MA, USA). These features included morphologic features such as perimeter, shape, size, and area. The statistical features of gray values included pixels, such as variance, gray average, and kurtosis. Texture features, such as correlation, entropy, contrast, homogeneity, inertia, and energy, which can be used to quantify intra-tumor heterogeneity, were calculated using the grayscale symbiosis matrix (GLCM) and gray level size zone matrix (GLSZM). A total of 204 imaging features representing qualitative breast image features were selected as the top imaging features through Mann–Whitney *U*-test and Pearson correlation coefficient. The JET color scale from MATLAB 2018a software (MathWorks, Natick, MA, USA) was applied to depict the discrepancies of the mammographic image grayscale.

### Pathological Analysis

We recorded the pathological data of the tumors, including histologic subtype, histological grade, and lymphatic metastasis. Immunohistochemical analysis of formalin-fixed paraffin-embedded tissue specimens was performed for the 43 TNBC patients who underwent breast cancer surgery. Standard biomarkers such as Ki-67 proliferation, estrogen receptor, progesterone receptor, epidermal growth factor receptor (EGFR), P53, and HER2 were reviewed in whole-tissue sample sections. The TIL levels of the surgical specimens of each patient which were stained with hematoxylin and eosin were reviewed by a pathologist with 20 years of experience in breast cancer diagnosis. The TIL levels were defined as the average percentage of lymphocyte infiltration per tumor and adjacent stroma and were reported at 10% increments. The following standards were complied with: (1) TILs should be evaluated within the boundaries of aggressive tumor. (2) TILs outside the tumor boundary and around the ductal carcinoma *in situ* (DCIS) and normal lobules should be excluded. (3) TILs in the tumor area with crush artifacts, necrosis, hyaline degeneration, and in the previous core biopsy site should be excluded. (4) The average TILs of the tumor area should be comprehensively evaluated by the pathologist. Hot spots should not be concentrated on. (5) All mononuclear cells (including lymphocytes and plasma cells) should be scored, but polymorphonuclear leukocytes should be excluded. Breast cancer with lymphocyte density >50–60% is currently called “lymphocyte-predominant breast cancer.” The tumor samples were divided into two groups: (1) the group with TIL levels below 50% was defined as the low-level TIL group; (2) the group with TIL levels higher than or equal to 50% were defined as the high-level TIL group.

### Statistical Analysis

To evaluate the differences in clinicopathologic characteristics between the low TIL and high TIL groups, categorical variables were analyzed by chi-square test or Fisher's exact test, and continuous variables were analyzed by *T*-test/ANOVA or Kruskal–Wallis test. Mann–Whitney *U*-test was used to compare the discrimination radiomics features between the low and high TIL groups, where appropriate. In addition, all radiological features were correlated with the clinicopathological characteristics of the patient and tumor using Pearson correlation coefficients. SPSS software (SPSS, version 25, Chicago, IL, USA) was used for all statistical analyses, and *p* < 0.05 was considered statistically significant.

## Results

**Figure 1:**
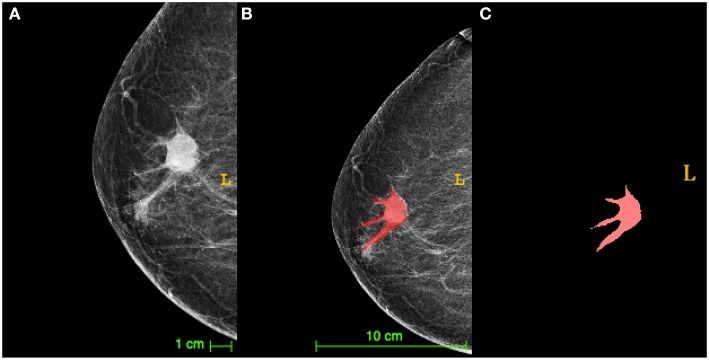
The diagram of triple-negative breast cancer (TNBC) lesions segmented by mapping the area of interest [region of interest (ROI)]. **(A)** A craniocaudal (CC) X-ray image of TNBC with the tumor (arrow) surrounded by lobulated projections and burrs. **(B)** The segmentation image of the tumor from **(A)** presented an irregular tumor shape. **(C)** The manual segmentation by drawing an ROI on the tumor in the same image as **(A)** in red was extracted *via* ITK-SNAP software.

TNBC lesions were segmented by mapping the area of interest (ROI) on the breast tumor, as shown in Figure 1. Table 1 shows the clinicopathologic date. Of the 43 patients, 32 (74.4%) exhibited low TIL levels, and 11 (25.6%) showed high TIL levels. The ages of patients ranged from 24 to 87 (mean age, 52.3) years. The patients in the high TIL group (mean age, 54.8 years) were older than those in the low TIL group (mean age, 51.6 years) (*p* = 0.534), but the differences were not statistically significant. All tumors were invasive ductal carcinoma, and patients in the low TIL groups were likely to have higher histological grade than those in the high TIL group [27/32 (84.4%) and 6/11 (54.5%)] (*p* = 0.043). The Ki-67 proliferation of the 26 patients was >14%. The Ki-67 negative threshold level in the high TIL group was lower than that in the low TIL group, and the difference between the two groups was statistically significant (*p* = 0.017).

**Table 1:** Patients and tumor clinicopathologic characteristics.

**Variables**	**Number of patients**	**Low TIL levels**	**High TIL levels**	***p-*value**
		**(<50%)**	**(>50%)**	
	**(*n* = 43)**	**(*n* = 32)**	**(*n* = 11)**	
Patients (*n*)	43	32	11	
Patient age, years (mean ± SD)	52.3 ± 14.4	51.6 ± 13.6	54.8 ± 17	0.534
Lymph node metastasis				
Negative	21 (48.8%)	16 (50%)	5 (45.5%)	0.795
Positive	22 (51.2%)	16 (50%)	6 (55.5%)	
Histologic grade				
Low	0	0	0	
Moderate	11 (25.6%)	5 (15.6%)	5 (45.5%)	0.043
High	32 (74.4%)	27 (84.4%)	6 (54.5%)	
Ki-67				
Low (<14%)	17 (39.5%)	16 (50%)	10 (9.0%)	0.017
High (>14%)	26 (60.5%)	16 (50%)	1 (91.0%)	
EGFR				
Negative	28 (65.1%)	22 (68.8%)	6 (54.5%)	0.394
Positive	15 (34.9%)	10 (31.2%)	5 (45.5%)	
P53				
Negative	16 (37.2%)	13 (40.6%)	3 (27.3%)	0.340
Positive	27 (62.8%)	19 (59.4%)	8 (72.7%)	

*EGFR, epidermal growth factor receptor; SD, standard deviation; TIL, tumor-infiltrating lymphocyte*.

**Figure 2:**
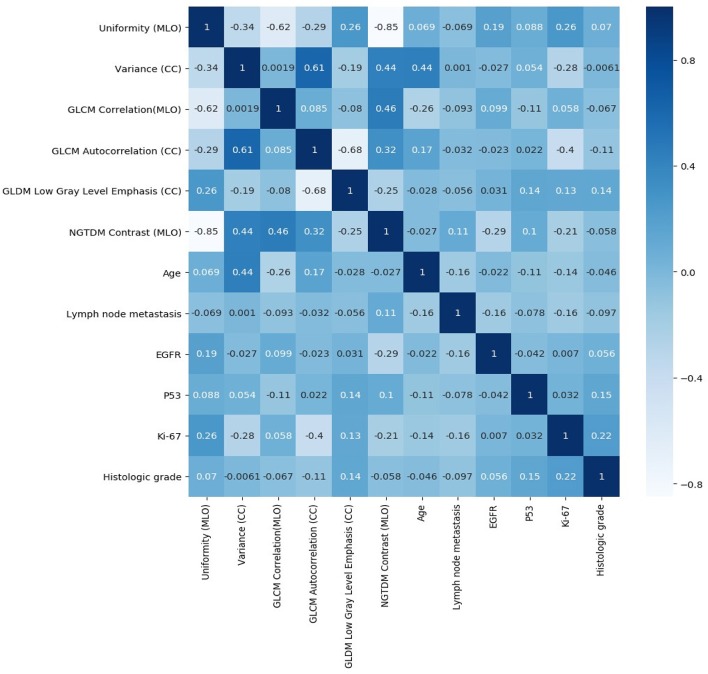
Pearson correlation coefficient heat map of mutual analysis between six top-class radiomics features (square lattice area of upper left corner) and mutual analysis between clinicopathologic characteristics and radiomics features (other area of square lattices). The values in the square lattices represent the magnitude of R value of correlation analysis displayed by color difference meanwhile.

**Figure 3:**
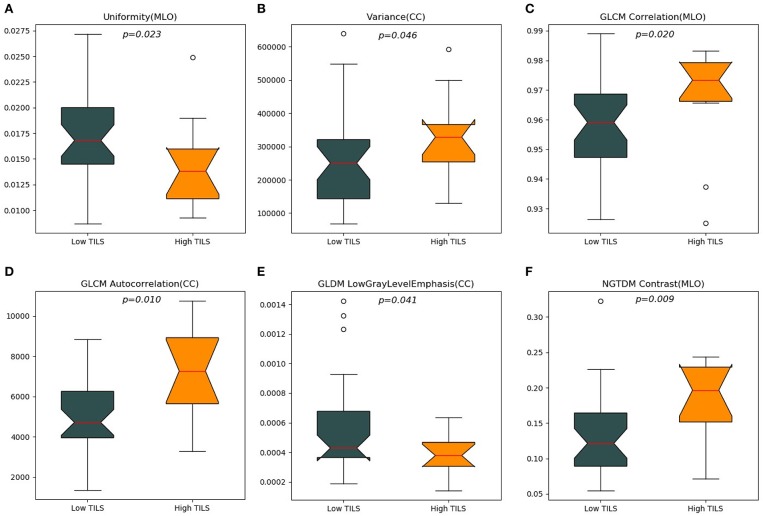
The top six ranked radiomics imaging characteristics chosen from craniocaudal (CC) and mediolateral oblique (MLO) view images. **(A)** View uniformity (MLO), **(B)** view variance (CC), **(C)** grayscale symbiosis matrix (GLCM) correlation (MLO), **(D)** GLCM autocorrelation (CC), **(E)** gray level difference matrix (GLDM) low gray level emphasis (CC), **(F)** neighborhood gray-tone difference matrix (NGTDM) contrast (MLO).

**Figure 4:**
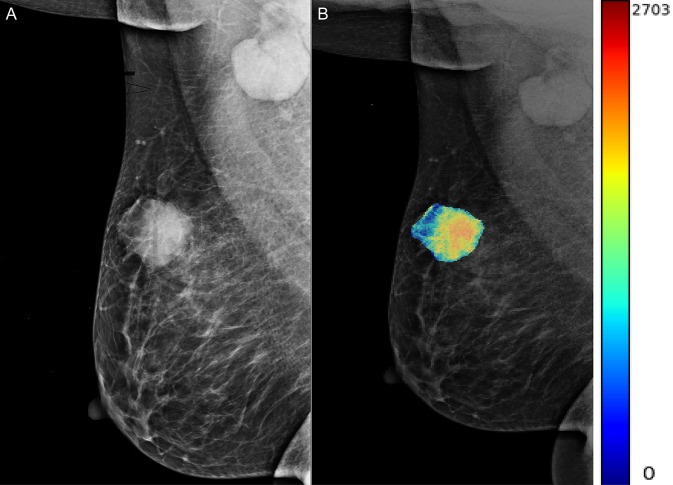
The woman, 56 years old, had triple-negative breast cancer, indicating a high tumor-infiltrating lymphocyte level in her right breast (arrow). **(A)** Right mediolateral oblique X-ray shows an uneven and smooth mass in the right breast. **(B)** X-ray image of mass density color overlay, showing uneven and smooth mass.

**Figure 5:**
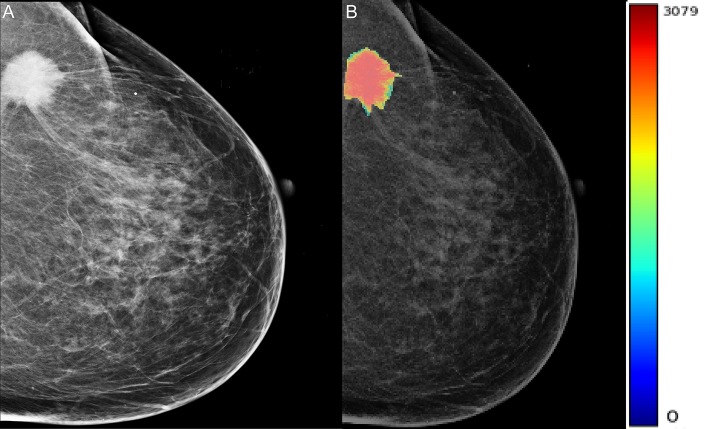
The woman, 60 years old, had triple-negative breast cancer, indicating low levels of neoplastic infiltrating lymphocytes in her left breast. **(A)** Oblique X-ray of the left mid lateral shows lobulated and partial burr mass of the left breast (arrow). **(B)** The X-ray image with a color overlay map of mass density indicates mass uniform and unsmooth.

A total of 204 features were extracted, and the selected lesions were normalized on CC and MLO. Fifty features (*p* < 0.05) were selected through the Mann–Whitney *U*-test. According to the most important characteristics selected by the Pearson correlation coefficient (Figure 2, square lattice area of upper left corner), six top-class features were screened out (Table 2), including uniformity (MLO) (*p* = 0.023), variance (CC) (*p* = 0.046), GLCM correlation (MLO) (*p* = 0.020), GLCM autocorrelation (CC) (*p* = 0.010), GLDM low gray level emphasis (CC) (*p* = 0.041), and NGTDM contrast (MLO) (*p* = 0.009) (Figures 3A–F). Figures 4, 5 show that tumors in the high TIL groups had a more non-uniform density and a smoother gradient of tumor patterns than those in the low TIL groups as observed in the mammographic images.

**Table 2:** Analysis of radiomics features between low and high TIL levels.

**Radiomics features**	**Low TIL levels**	**High TIL levels**	***p*-value**
	**(<50%)**	**(>50%)**	
	**(*n* = 32)**	**(*n* = 11)**	
Uniformity (MLO)			
Mean	0.017	0.014	0.023
Range	0.009–0.027	0.009–0.025	
Variance (CC)			
Mean	260,776.234	328,611.123	0.046
Range	69,059.883–638,614.685	129,976.869–592,881.928	
GLCM correlation (MLO)			
Mean	0.959	0.967	0.020
Range	0.926–0.989	0.925–0.983	
GLCM autocorrelation (CC)			
Mean	5,032.505	7,170.002	0.010
Range	1,367.669–8,852.751	3,287.865–10,759.123	
GLDM low gray level emphasis (CC)			
Mean	0.00057	0.00038	0.041
Range	0.00019–0.0014	0.00014–0.00063	
NGTDM contrast (MLO)			
Mean	0.133	0.180	0.090
Range	0.054–0.323	0.071–0.244	

*CC, craniocaudal; GLCM, grayscale symbiosis matrix; GLDM, gray level difference matrix; MLO, mediolateral oblique; NGTDM, neighborhood gray-tone difference matrix; TIL, tumor-infiltrating lymphocyte*.

The significance testing and Pearson correlation coefficient of clinicopathologic characteristics, age, and radiomics features are listed in Table 3 and Figure 2. Ki-67 was significantly correlated with uniformity, variance, and GLCM autocorrelation (*p* = 0.03, r = 0.26; *p* = 0.006, r = −0.28; and *p* = 0.005, r = −0.4, respectively). Age was significantly correlated with variance and GLCM (*p* = 0.007, r = 0.44 and *p* = 0.03, r = −0.26, respectively). EGFR and NGTDM contrast significantly differed among the radiomics features (*p* = 0.04, r = −0.29).

**Table 3:** The correlation analysis of clinicopathologic characteristics and radiomics features.

**Features**	**Uniformity (MLO)**	**Variance (CC)**	**GLCM correlation (MLO)**	**GLCM autocorrelation (CC)**	**GLDM low gray level emphasis (CC)**	**NGTDM contrast (MLO)**
Ki-67						
*p*-value	0.0303^*^	0.0062^*^	0.4950	0.0058^*^	0.1757	0.1240
r-value	0.26	−0.28	0.058	−0.4^*^	0.13	−0.21
Histologic grade						
*p*-value	0.2406	0.2875	0.2778	0.3813	0.3813	0.2588
r-value	0.07	−0.0061	−0.067	−0.11	0.14	−0.058
Lymph node metastasis						
*p*-value	0.4758	0.4087	0.2149	0.4758	0.4855	0.2923
r-value	−0.069	0.001	−0.093	−0.032	−0.056	0.11
Age						
*p*-value	0.1732	0.0070^*^	0.0342^*^	0.0519	0.2278	0.1732
r-value	0.069	0.44	−0.26	0.17	−0.028	−0.027
EGFR						
*p*-value	0.0647	0.4043	0.3750	0.4443	0.2338	0.0404^*^
r-value	0.19	−0.027	0.099	−0.023	0.031	−0.29
P53						
*p*-value	0.4450	0.4057	0.3485	0.3767	0.3767	0.3672
r-value	0.088	0.054	−0.11	0.022	0.14	0.1

* *Significant differences*.

*EGFR, epidermal growth factor receptor; CC, craniocaudal; GLCM, grayscale symbiosis matrix; GLDM, gray level difference matrix; MLO, mediolateral oblique; NGTDM, neighborhood gray-tone difference matrix*.

## Discussion

Studies have proved that TIL levels have a strong prognostic value, which can improve the distant recurrence-free survival, disease-free, and overall survival estimates for TNBC patients treated with adjuvant/neoadjuvant chemotherapy (22, 23). Increased TIL levels have been observed to be positively correlated with prolonged survival and increased pathological complete response rates (24–26). Because of the uneven distribution of TILs within the tumor, TIL levels obtained by biopsy in a specific part of the tumor may not reflect the entire tumor. We employed a radiomics approach to observe the correlation of tumor TIL levels and quantitative imaging characteristics of digital mammography in TNBC patients. The results of the present study suggest that there are differences in the clinicopathological features of TNBC and mammography with respect to TIL levels.

Several previous studies have reported the relationship between TIL levels and MRI findings for TNBC patients (18, 27). However, no studies have investigated the relationship between TIL levels and digital mammographic images. In our study, we analyzed whether quantitative digital mammographic image features have a similar correlation effect. MRI and mammography show differences in underlying imaging characteristics, but in our study, we analyzed the mammographic image data for only breast morphology, density, or anatomical characteristics for evaluating breast cancer TILs in terms of imaging characteristics.

TNBC is known to be more invasive and exhibits poorer results. Early identification of TNBC from other subtypes of breast cancer is crucial and can help clinicians build an ideal treatment strategy before final pathologic confirmation. Radiomics is likely to play an important role in the detection of breast cancer and monitoring the development and treatment response. In this study, we found that six radiomics features were identified as most significant variables of tumor TILs: uniformity, variance, GLCM correlation, GLDM low gray level emphasis, NGTDM contrast, and GLCM autocorrelation.

The measure of the sum of squares of each intensity value denotes uniformity, which is a measure of the uniformity of an image array; greater uniformity implies greater uniformity or a smaller range of discrete intensity values. Variance represents the mean of the squared distances from the mean of each intensity value, which is the mean distribution of measurements. Correlation can be expressed by the value between 0 (uncorrelated) and 1 (perfectly correlated), which represents the linear dependence between the gray value and the corresponding voxel in GLCM and represents the smooth gradient of the pattern in the quantitative image. Autocorrelation is a measure of the size of texture fineness and roughness. A measure of the distribution of low gray levels indicates that the higher the value, the greater the concentration of low gray values in the image, which represents the brightness in the mammographic image. Contrast is a method of measuring spatial intensity variation, which also depends on the entire dynamic grayscale range. When the dynamic range and the rate of spatial change are high, the contrast is high. Based on the above explanation (28), our study shows that the high TIL levels may be more uneven than low intensity values; the high TIL levels may be smoothed by the gradient pattern, and high TIL levels may be denoted by regions brighter than the gray values which are lower than the level of the mammary gland image. A previous study showed that TNBC was more uneven on dynamic contrast-enhanced MRI (29). Although the imaging modes used were different compared with mammographic images, it may also have the same effect on the radiomics feature outcome. The significance tests of Ki-67, EGFR, radiomic characteristics, and Pearson correlation coefficient were statistically significant (*p* < 0.05), suggesting that the high expressions of Ki-67 and EGFR have uniform intensity values and dynamic grayscale ranges in the mammographic image.

The clinicopathology of tumors can reflect tumor biology and affect the outcome of chemotherapy in TNBC patients (30–33). Our study found that the TNBC histological grade of the high TIL group was lower than that of the low TIL group (84%). These findings are consistent with the results of Ku et al. (27). These results indicated that tumor with low TIL levels grows rapidly and has a high tumor necrosis rate. The proliferation rate of Ki-67 in 26 patients was more than 14%, and the difference between the two groups was statistically significant (*p* = 0.017). The tumor proliferation of breast cancer patients can be reflected by Ki-67 expression (34). Particularly, if the level is ≥14%, the Ki-67 level is positive, and if the level is <14%, the Ki-67 level is negative (35). The Ki-67 marker index is considered an important prognostic marker and a significant indicator of potential triage to chemotherapy (36). In this study, we found that the high TIL group had a more negative threshold Ki-67 level (<14%), and this result verified that TNBC patients with high TIL levels probably have low Ki-67 levels. Then, the tumors have less malignant cell proliferation, and they exhibit a positive reaction to neoadjuvant chemotherapy (37, 38).

This study had some limitations. First, this retrospective study only obtained single-vendor images from single institutions, which may have limited the universality of the findings. In addition, the generalizability of the findings to other vendors of the image needs to be verified. Second, the number of patients included was very small, thus affecting the statistical significance of the data. Therefore, further analysis of larger cohort studies may provide other variables that are significantly associated with TIL levels in TNBC patients. Third, most radiomics features that differed between the two groups were not statistically significant. Therefore, we need to generalize our results through validation studies in the future. Finally, because of the lack of MRI data, we could not compare the performance of mammograms and DCE-MR images of this population. However, testing and comparing the relationship between radiological features of mammograms and TIL levels in TNBC patients is an important follow-up study. Mammography is the most used routine breast cancer screening and diagnostic method. If automatic radiomics features are validated for analysis of TIL levels, more information can be provided from mammograms to assist radiologists and clinicians to diagnose and treat TNBC.

In conclusion, quantitative imaging radiomics features from digital mammograms were found to be a useful method for discriminating low and high TIL levels in patients with TNBC. Research needs to be conducted on a larger scale to assess these findings and examine their relevance to the radiological features of DCE-MRI of the breast in the future.

## Data Availability Statement

All datasets generated for this study are included in the article/supplementary material.

## Ethics Statement

This retrospective study was approved by the Institutional Ethics Review Committee of the China-Japan Friendship Hospital and the informed consent requirements were obtained from all patients.

## Author Contributions

The acquisition, analysis, data explanation, and manuscript draft were finished by HY. XM and JF are responsible for the analysis and explanation of the radiomics imaging features data. HC analyzed and explained the pathological analysis. XH, WG, LD, YC, YW, and XL acquired the clinical information and revised the manuscript. LZ, JY, and GM designed the study and made multiple revisions to the manuscript.

### Conflict of Interest

The authors declare that the research was conducted in the absence of any commercial or financial relationships that could be construed as a potential conflict of interest.
